# Characterization of Antineoplastic Agents Inducing Taste and Smell Disorders Using the FAERS Database

**DOI:** 10.3390/ph17091116

**Published:** 2024-08-23

**Authors:** Risa Hamazaki, Yoshihiro Uesawa

**Affiliations:** Department of Medical Molecular Informatics, Meiji Pharmaceutical University, Tokyo 204-8588, Japan

**Keywords:** taste disorder, olfaction, chemotherapy, cancer

## Abstract

Taste and smell disorders can worsen the nutritional status of patients receiving chemotherapy and potentially affect drug treatment. However, there is limited knowledge on antineoplastic agents that induce taste and smell disorders. Therefore, we used the U.S. Food and Drug Administration Adverse Event Reporting System database to analyze the characteristics of patients and antineoplastic agents in relation to taste and smell disorders. No gender differences related to the onset of taste and smell disorders were found, whereas older age was identified as a possible risk factor. Among the antineoplastic agent classes, protein kinase inhibitors appeared to be particularly likely to induce taste and smell disorders. According to the cluster and principal component analyses, antineoplastic agents were deemed to induce taste or smell disorders. In addition, antineoplastic agents that decreased or changed these sensations could be classified. These findings might be useful in selecting drugs for patients experiencing similar side effects.

## 1. Introduction

Taste and smell disorders caused by antineoplastic agents can decrease food intake in patients undergoing chemotherapy [1,2]. Meanwhile, malnutrition in patients with cancer can lead to weight loss, prolonged treatment, and reduced quality of life [1,3,4,5]. A study using the Italian spontaneous adverse drug reaction (ADR) reporting database identified 182 cases of taste and/or smell impairments. In particular, 75.3% of patients reported taste alterations alone, 11.0% reported smell impairments alone, and 13.7% reported that both taste and smell alterations occurred [6]. The mechanisms by which pharmaceuticals alter taste and smell are not completely understood, but multiple mechanisms have been suggested [7]. Mechanisms for the development of taste and smell disorders include effects on receptors [8], drug secretion into saliva, and dryness of the oral cavity [9]. In a comprehensive search of all drugs using a large adverse drug reaction reporting database, a recent study identified antineoplastics and immunomodulatory drugs as frequently reported drug classes with respect to general gustatory and olfactory ADRs [10]. However, our knowledge of antineoplastic agents that induce these side effects is limited, and further research is required. A dedicated analysis of antineoplastic agents that induce taste and smell disorders might provide useful information on drug therapy for patients with cancer. In addition, because it is difficult for patients to distinguish taste disorders from smell disorders, patients with an abnormal sense of smell might also experience an abnormal sense of taste [11,12]. The U.S. Food and Drug Administration (FDA) Adverse Event Reporting System (FAERS) database has a large number of reports because it collects reports from many countries, thereby permitting the analyses of infrequent or underreported side effects. Using the FAERS database, this study aimed to understand or characterize antineoplastic agents that induce taste and smell disorders.

## 2. Results

### 2.1. Creation of Data Tables

**Figure 1:**
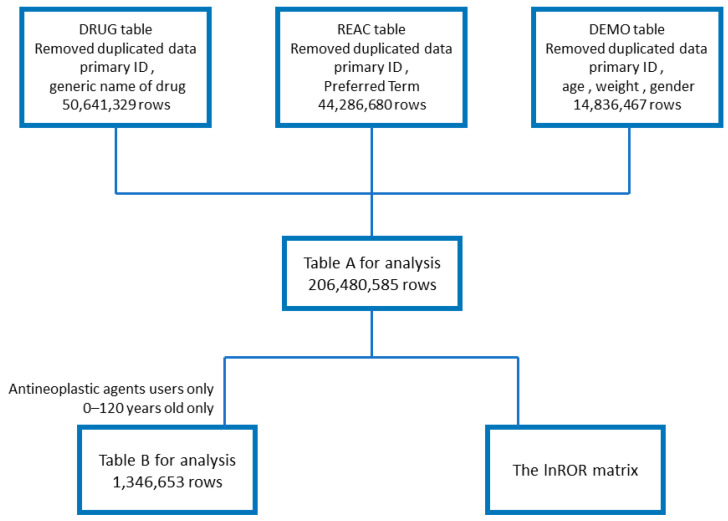
Flowchart for creating tables for analysis.

The analysis used a data table that combined the FAERS drug information (DRUG) table (50,641,329 rows), adverse reaction information (REAC) table (44,286,680 rows), and patient demographic information (DEMO) table (14,836,467 rows). Duplicate content was removed during data table creation. The flowchart of table creation is presented in Figure 1.

Table A for analysis was created by de-duplicating each of the three tables (DRUG, REAC, and DEMO tables) and combining them by using the primary ID. Table B for analysis and the lnROR matrix table were created from Table A. Table A was used to study the number of reports of taste and smell disorders and calculate the *p*-values and reporting odds ratios (RORs) for the drugs and ADRs. Table B presents information on the gender and age of patients who developed taste and smell disorders after antineoplastic agent treatment. The lnROR matrix was used for hierarchical cluster and principal component analyses.

### 2.2. Number of Reports of Taste and Smell Disorders

The data tables for analysis included 18,764 reports of abnormal taste and 1470 reports of abnormal olfaction among patients who used antineoplastic agents. There were 892 reports of combined taste and smell abnormalities (Table 1).

### 2.3. Relationships of Patient Age and Gender with Taste and Smell Disorders Induced by Antineoplastic Agents

Table 2 presents data on the gender and age of the patients who used antineoplastic agents. The results revealed no significant association with gender, whereas the risk of antineoplastic agent-induced taste and smell disorders was higher in patients aged ≥70 years.

### 2.4. Identification of Antineoplastic Agents That Induce Taste and Smell Disorders

**Figure 2:**
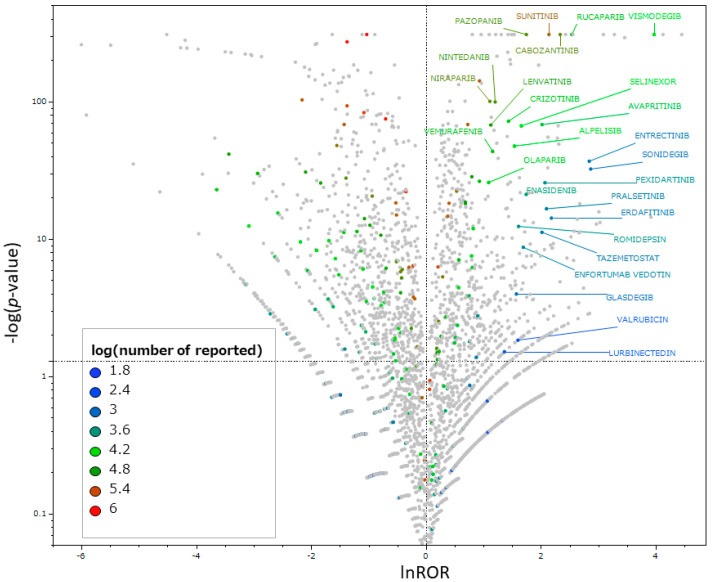
Volcano plot for the antineoplastic agents and taste and smell disorders. The vertical axis presents the statistical significance based on Fisher’s exact test, and the horizontal axis presents the risk of inducing taste and smell disorders. Drugs significantly associated with adverse reactions are indicated in the upper right corner. The scatterplot is colored for antineoplastic agents according to the number of drug reports. Non-antineoplastic agents are presented in gray, and antineoplastic agents with lnROR ≥ 1 and −log*p* ≥ 1.3 or greater are labeled with the drug name. Drugs with 100 or more reports are presented.

Scatter plots were created to identify antineoplastic agents that increased the risk of taste and smell disorders. As presented in Figure 2, 56 drugs were found to potentially induce taste and smell disorders based on their RORs and *p*-values (Supplementary Table S1).

### 2.5. Relationships between the Class of Antineoplastic Agents and Taste and Smell Disorders

Next, the risk of taste and smell disorders was examined by the Anatomical Therapeutic Chemical (ATC) class. In particular, the percentage of antineoplastic agents that were likely to induce taste and smell disorders in each of the seven categories of antineoplastic agents was calculated (Table 3). Based on an analysis using Fisher’s exact test, protein kinase inhibitors and other antineoplastic agents were found to significantly increase the risk of taste and smell disorders among the antineoplastic agents (Table 4) (Supplementary Table S2).

### 2.6. Hierarchical Cluster Analysis

**Figure 3:**
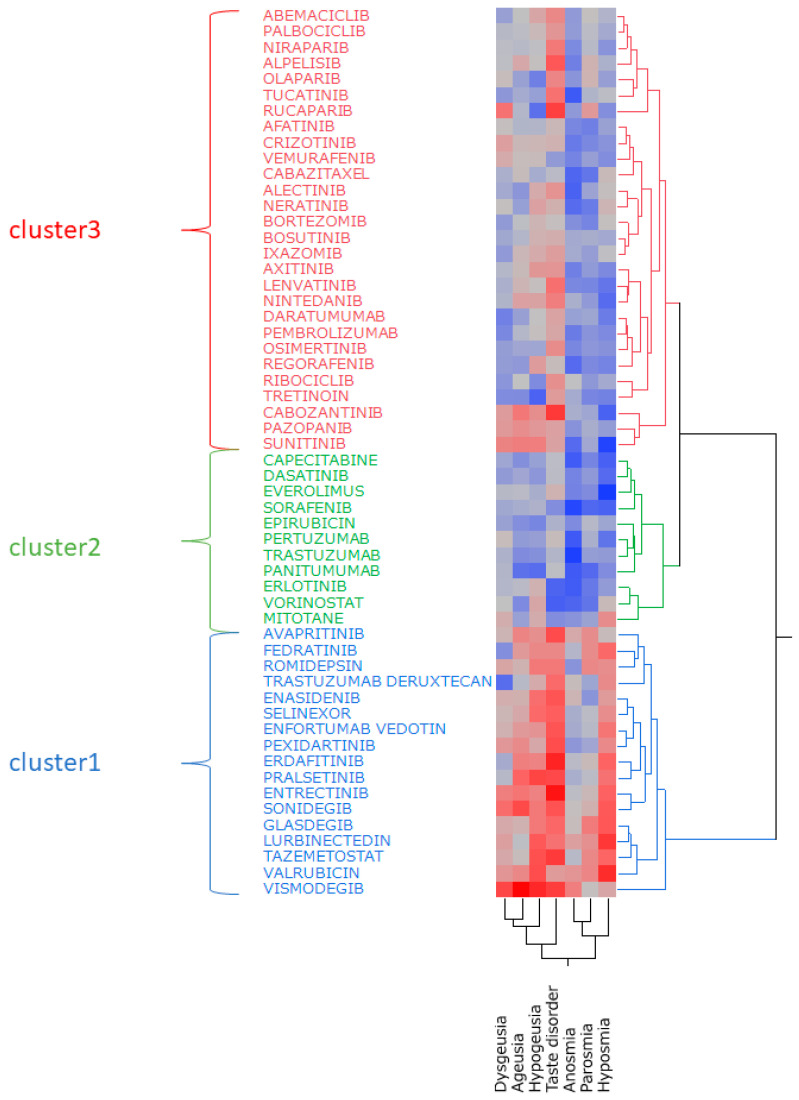
Hierarchical cluster analysis. The figure presents the relationship between seven side effects related to taste and smell disorders and 56 antineoplastic agents. In the color map, red indicates positive correlations, and blue indicates negative correlations.

**Figure 4:**
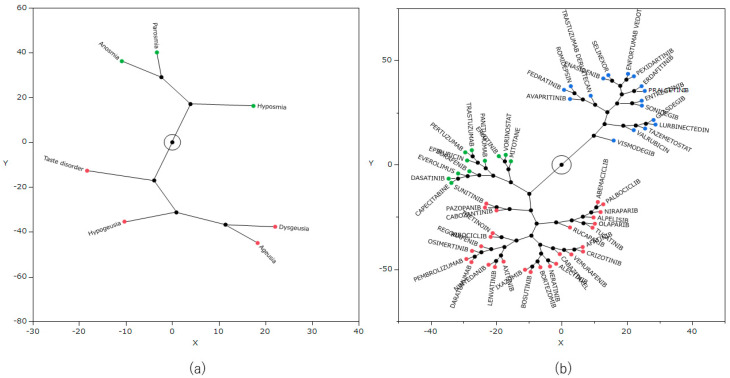
Constellation plot. The figure provides a visual understanding of the results of the cluster analysis. Dots indicate individual data lines, and line lengths indicate relative distances between clusters. Plots (**a**) and (**b**) are related to the side effects and drugs, respectively.

Hierarchical cluster analysis was performed using the natural logarithm of the ROR (lnROR) matrix of side effects and antineoplastic agents, which identified three clusters (Figure 3). In addition, from the results of the cluster analysis, a constellation plot of drugs and side effect words was created (Figure 4).

### 2.7. Principal Component Analysis

**Figure 5:**
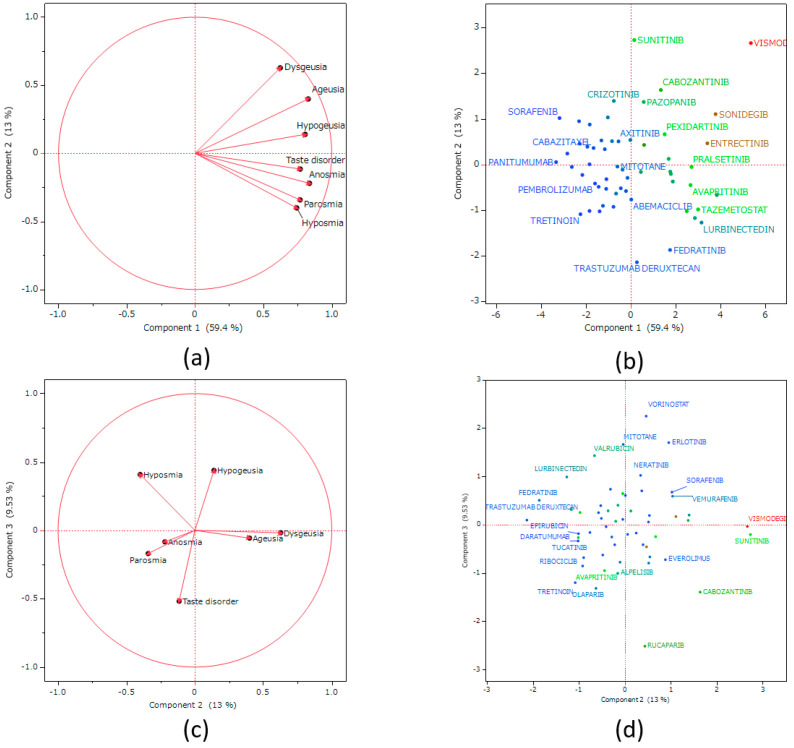
Relationships of taste and smell disorders with antineoplastic agents using principal component analysis. Loadings plots (**a**) and (**c**) present the association between adverse events related to taste and smell disorders and each principal component. Each loading vector represents an adverse effect. Score plots (**b**) and (**d**) present the relationship between antineoplastic agents and each principal component. Each dot represents an antineoplastic agent. Each plot is colored by lnROR based on the calculations for all drugs.

**Figure 6:**
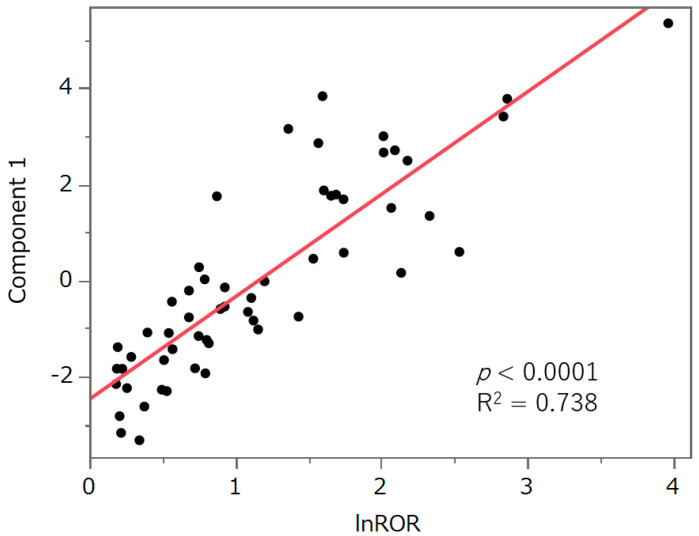
Bivariate relationship between principal component 1 and lnROR.

Figure 5 presents the results of the principal component analysis. The contributions of principal components 1, 2, and 3 were 59.4%, 13%, and 9.53%, respectively. Principal component 1 was positively correlated with lnROR in Section 2.4. (*p* < 0.0001, R^2^ = 0.738, Figure 6). Principal component 2 was positively correlated with the preferred terms (PTs) dysgeusia (*p* < 0.0001) and ageusia (*p* = 0.0023), and negatively correlated with parosmia (*p* = 0.0099) and hyposmia (*p* = 0.0022). Principal component 3 was positively correlated with the PTs hypogeusia (*p* = 0.0007) and hyposmia (*p* = 0.0018), and negatively correlated with taste disorder (*p* < 0.0001).

The correlation between principal component 1 and “lnROR based on calculations for all drugs” was determined, and a least-squares line was fitted.

## 3. Discussion

### 3.1. Number of Reports of Taste and Smell Disorders

Among the patients listed in Table A who developed taste or smell disorders following antineoplastic agent exposure, approximately 92.4% of the patients only developed a taste disorder, and approximately 3.0% only developed a smell disorder. Approximately 4.6% of patients developed both taste and smell disorders.

In this study, more disorders related to the sense of taste were reported than those related to the sense of smell, consistent with previous findings described in Section 1 [6].

### 3.2. Characteristics of Patients with Taste and Smell Disorders and Antineoplastic Agents

This study identified no difference in the risk of antineoplastic agent-induced taste and smell disorders according to gender. Studies on the relationship of gender with this risk have been inconsistent. Some studies identified female gender as a risk factor for the development of taste disorders [13,14], whereas others reported no significant differences regarding alterations in taste and smell between men and women undergoing chemotherapy [15].

Conversely, older age was identified as a risk factor for taste and smell disorders. Similar to that noted for gender, the association between age and the risk of taste and smell disorders has been inconsistent. Some studies have suggested that young people have a higher risk of taste and smell alterations [16,17], whereas others found that older age increased the risk [13,18,19].

Concerning drug-induced taste disorder, a prior study reported a significantly higher incidence among elderly patients [20]. This suggests that the risk of dysgeusia induced by antineoplastic agents increases with advancing age, as is usually the case in patients with a drug-induced taste disorder. To confirm the findings of this study, it would be ideal to conduct observational studies (especially cohort or case–control studies), and if possible, the obtained results could be complemented by randomized controlled trials. Based on the study results, the appropriate monitoring of taste and smell should be conducted when administering antineoplastic agents to elderly patients.

### 3.3. Relationships of Antineoplastic Agents with Taste and Smell Disorders

A volcano plot was created, and 56 antineoplastic agents were found to likely induce taste and smell disorders. Some of the identified drugs, such as daratumumab, nintedanib, epirubicin, and tretinoin, did not list taste and smell disorders as side effects in their Japanese package inserts or in the FDA’s relevant drug information [21,22]. In addition, a pseudo-positive result might have been obtained for tretinoin. Caution should be exercised in the interpretation of the detected drugs.

### 3.4. Relationship between the Class of Antineoplastic Agents and Taste and Smell Disorders

The results indicated that protein kinase inhibitors represent the most likely drug class to induce taste and smell disorders among antineoplastic agents. A prior study using the Italian database of spontaneous ADR reports similarly suggested that protein kinase inhibitors were associated with taste and smell impairment [6]. Studies on the association between protein kinase inhibitors and alterations in taste and smell suggested that oral toxicities, such as oral mucositis and xerostomia, affect taste receptor cells and olfactory receptor neurons, and neurodegeneration might be involved in the mechanism of taste and smell disorders [23]. Decreased Wnt-β catenin signaling can affect taste by affecting cell differentiation. Sunitinib, a signal for which was detected in this study, could have a similar effect [23,24]. EGFR inhibitors such as erlotinib, afatinib and osimertinib, FGFR inhibitors such as erdafitinib, and the VEGFR inhibitor axitinib can also affect β-catenin signaling [23,25,26].

In addition, the “other antineoplastic agents” class was significantly more likely to induce taste and smell disorders. Hedgehog pathway inhibitors are believed to affect taste by inhibiting the differentiation of taste cell precursors into taste cells [23,27,28]. In this study, three drugs classified as hedgehog pathway inhibitors (L01XJ) in the ATC classification, namely vismodegib, sonidegib, and glasdegib, were detected as drugs that can cause this taste disorder. However, the category “other antineoplastic agents” includes a wide variety of drugs. Tretinoin, which was included in this class in this study, was found to have a significant link to taste disorders (*p* < 0.05, ROR > 1) with more than 100 reported cases. However, tretinoin is a vitamin A derivative that is used for various purposes other than cancer treatment. Vitamins are sometimes used to treat smell disorders, and some studies [29] have suggested that topical vitamin A can improve smell disorders. Therefore, pseudo-positives are possible, and caution should be exercised in interpreting these medications. The appropriate monitoring of taste and smell should be conducted when administering the antineoplastic agents that were estimated in this analysis to be likely to induce taste and smell disorders. Such actions might permit the early detection of adverse effects and timely intervention.

### 3.5. Hierarchical Cluster Analysis

Hierarchical cluster analysis is a method of grouping similar data to generate a classification [30]. The result of this analysis classified the drugs into three clusters (Figure 3), and Table 5 lists each cluster and the included drugs. Cluster 1 displayed a strong correlation with many of the seven side effect words, and the cluster included drugs such as vismodegib and tazemetostat. Cluster 3 exhibited a positive correlation for some side effects, whereas cluster 2 displayed a negative correlation with most side effects. All of the drugs used in this cluster analysis were considered likely to induce taste and smell disorders. However, the type of side effects that are likely to occur among them can vary by drug.

Based on these results, we further clarified the detailed characteristics of antineoplastic agents that induce taste and smell disorders by performing principal component analysis.

### 3.6. Principal Component Analysis

Principal component analysis reduces the dimensionality of the dataset, thereby increasing the potential for interpretation while reducing information loss [31]. Principal component 1 was positively correlated with lnROR (Figure 6). Principal component 1 represents a risk of taste and smell disorders. Principal component 2 was positively correlated with the PTs dysgeusia and ageusia, which are related to the sense of taste, and was negatively correlated with parosmia and hyposmia, which are related to the sense of smell. This suggests that principal component 2 can be plotted in a more positive direction as it relates to the taste side effects, and in a more negative direction as it relates to the smell side effects. Principal component 3 was positively correlated with the PTs hypogeusia and hyposmia, which are related to decreases in chemosensory perception. This component was also negatively correlated with the PT taste disorder, which is related to chemosensory changes. From this result, principal component 3 can be plotted in a positive direction for side effects that decrease chemosensory perception, and in a negative direction for side effects that change sensory perception. Based on this speculation, we confirmed the score plots (Figure 5) for principal components 2 and 3. Erlotinib might be the drug likely to cause a decrease in taste, cabozantinib might be the drug likely to cause a change in taste, ribociclib might be the drug likely to cause a change in smell, and lurbinectedin might be the drug likely to cause a decrease in smell. These inferences are expected to be useful in monitoring side effects in patients using antineoplastic agents.

### 3.7. Limitations

This study had several limitations. First, FAERS is a spontaneous reporting database of ADRs. Spontaneous reporting of ADRs is subject to reporting bias [32,33] including underreporting and missing or erroneous data. In addition, because the denominator of the drug users is unknown, the true incidence rates cannot be calculated, making it impossible to perform an absolute risk assessment. As a countermeasure, we deleted data considered to be missing or erroneous in the analysis data tables for age and gender. In addition, the number of drugs detected was limited by the number of reports.

In addition to underreporting, other biases have been recognized. The notoriety effect [34] describes an increase in the number of reports of adverse events that have become the focus of attention. The ripple effect [34] refers to the increase in reports on drugs of the same type and efficacy as the specific drug that was the focus of adverse event monitoring. The Weber effect [35] describes the pattern in which the number of reports increases immediately after marketing and then decreases over time. These biases may be related to the underestimation or overestimation of reporting. In addition, another bias is the masking effect, in which certain adverse events are underestimated because of the association of other drugs [36]. Another concern is that when multiple drugs are administered, it is difficult to identify the drug responsible for the adverse reaction [37,38]. Furthermore, information such as the primary disease, concomitant medications and the number of medications, the administration method, and the duration of drug administration, which were not included in this analysis, could be confounding factors that influence the occurrence of adverse effects. We hope that future studies will provide findings considering these factors.

## 4. Materials and Methods

### 4.1. FAERS Database

To comprehensively analyze taste and smell disorders induced by antineoplastic agents, we used the FAERS database, which contains cases from January 2004 to March 2022 (May 2022 public version) [39]. FAERS is a large FDA-published database of spontaneous adverse drug reaction reports. FAERS consists of seven data tables: DEMO, DRUG, REAC, Outcome (OUTC), Report Sources (RPSR), Indication (INDI), and Therapy (THER). Each table can be joined. The DEMO table contains basic patient information such as age, gender, and weight. The DRUG table contains drug information such as the drug name and administration method. The REAC table contains adverse event information such as PTs. The OUTC table contains outcome information. The RPSR table contains information sources. The INDI table contains drug indication information. The THER table contains information on the duration of treatment. The curation of generic names of drugs were conducted by INTAGE Healthcare Inc. (Tokyo, Japan, https://www.intage-healthcare.co.jp/, accessed on 31 June 2024). For this analysis, we used Table A for analysis, which contains a combination of the DRUG, REAC, and DEMO tables with the elimination of duplicates. The combining was performed by primary ID.

As this study used anonymized data from an open access database, the requirement for ethical approval and informed consent by the Meiji Pharmaceutical University Ethics Committee was waived.

### 4.2. Adverse Event Terms and Drugs for Analysis

The analyzed drugs were all reported to FAERS. Antineoplastic agents were defined as drugs classified as antineoplastic agents (L01) based on the ATC classification, a classification system proposed by the World Health Organization that categorizes drugs according to their therapeutic effects and characteristics [40]. Regarding adverse events, we used the Standardized MedDRA Query (SMQ) [41] in MedDRA ver. 25.0, and 14 adverse events from “taste and smell disorders” (SMQ: 20000046, Table 6) were included in the analysis after excluding congenital anosmia. Specifically, the taste disorders were “dysgeusia”, “ageusia”, “taste disorder”, “hypogeusia”, “hallucination, gustatory”, “hypergeusia”, and “gustometry, abnormal”. The smell disorders were “anosmia”, “parosmia”, “hyposmia”, “hallucination, olfactory”, “olfactory nerve disorder”, “olfactory test abnormal”, and “olfactory dysfunction”.

### 4.3. Number of Reports of Taste and Smell Disorders

Table A was used to extract the number of taste and smell disorders in cases in which a drug classified as an antineoplastic agent (L01) in the ATC classification was used. The measurement results are listed in Table 1. Adverse event terms were defined using the 14 terms listed in Section 4.2. Patients receiving antineoplastic agents were counted as having developed an adverse reaction if they experienced at least one taste or smell disorder.

### 4.4. Relationships of Taste and Smell Disorders with Age and Gender Among Patients Using Antineoplastic Agents

We removed all cases other than those in which antineoplastic agents were used on the basis of the data table created in Section 4.1. In the case of multiple reports with the same primary ID, if there was at least one report of a taste or smell disorder, the patient was considered to have developed a taste or smell disorder. Cells in which age was not given in years were deleted. In addition, cases with ages lower than 0 or greater than 120 were removed as likely errors. The resulting table (Table B) contained 1,346,635 rows (Figure 1). Elderly persons were defined as those “70 years of age or older”. Patients were divided into two age groups: <70 and ≥70 years. Gender was categorized as male or female. We further divided the patients into two groups according to the presence or absence of taste and smell disorders and analyzed the results using Fisher’s exact test to identify significant correlations. The results are listed in Table 2.

### 4.5. Relationships between Antineoplastic Agents and Taste and Smell Disorders

Using Table A, a 2 × 2 contingency table was created (Table 7). A value of 0.5 was added to all cells as a Haldane correction [42,43,44]. RORs and *p*-values were calculated for the antineoplastic agents and side effect terms related to taste and smell disorders using Fisher’s exact test. We performed a relative evaluation by detecting the signal represented by the RORs of individual drugs. This imbalance analysis method allowed us to identify cases in which a high number of drug side effects were reported [45]. Based on the calculated values, a volcano plot was created with the vertical axis being the reciprocal of the common logarithm of the *p*-value (−log*p*) and the horizontal axis being lnROR, the natural logarithm of the ROR [46,47,48,49,50] (Figure 2). Volcano plots were used to visually interpret adverse drug events. This type of scatter plot is often used to understand trends in gene expression in microarray data analysis [51]. If the *p*-value is less than 1 × 10^−308^ power, it is not calculated by the statistical software JMP Pro 16.2, and thus, −log*p* was set to 308 as an approximation. Antineoplastic agents with statistically significant associations with adverse events (ROR > 1) [46,47,48,49,50,52] and more than 100 reports were selected as drugs for cluster and principal component analyses.

### 4.6. Relationships of the Class of Antineoplastic Agents with Taste and Smell Disorders

Based on the ATC classification, antineoplastic agents included alkylating agents (L01A), antimetabolites (L01B), plant alkaloids and other natural products (L01C), cytotoxic antibiotics and related substances (L01D), protein kinase inhibitors (L01E), monoclonal antibodies and antibody drug conjugates (L01F), and other antineoplastic agents (L01X). For each of these categories, we calculated the percentage of antineoplastic agents that are likely to induce taste and smell disorders. In addition, a 2 × 2 contingency table was created for each of the seven categories of antineoplastic agents as described in Section 4.5 (Table 7). The *p*-values were calculated using Fisher’s exact test to identify significant correlations.

### 4.7. Creation of Data Tables for Principal Component and Cluster Analyses

A table of the lnROR for each taste and smell disturbance-related adverse reaction to antineoplastic agents (lnROR matrix) was created (Figure 1). The drugs analyzed were antineoplastic agents significant at *p* < 0.05 and ROR > 1 with at least 100 reported cases, as selected in Section 4.5. Side effects with fewer than 3000 reports were removed. The seven PTs included in the analysis were dysgeusia, ageusia, taste disorder, anosmia, parosmia, hypogeusia, and hyposmia (Supplementary Table S3).

### 4.8. Hierarchical Cluster Analysis

A hierarchical cluster analysis was performed using the tally sheets created in Section 4.7. The Ward method was used for the calculation [30].

### 4.9. Principal Component Analysis

Similar to the hierarchical cluster analysis, a principal component analysis was performed using the tally sheets created in Section 4.7. A correlation coefficient matrix was used for the analysis focusing on principal components 1, 2, and 3.

### 4.10. Statistical Analysis

Statistical analyses were performed using JMP Pro 16.2 (SAS Institute Inc., Cary, NC, USA). The statistical significance level was set at *p* < 0.05.

## 5. Conclusions

Based on the results of this study, 56 antineoplastic agents were estimated to be likely to induce taste and smell disorders. The characteristics of each antineoplastic agent related to these side effects, “disorder of taste/smell” and “decrease/change of taste and smell” were also estimated by cluster and principal component analyses. We hope that these findings will be confirmed in future observational studies (especially cohort and case–control studies) and randomized controlled trials to obtain further detailed data on antineoplastic agents that induce taste and smell disorders. These findings will potentially be useful for selecting drugs and monitoring side effects in patients experiencing similar side effects.

## Figures and Tables

**Table 1:** Number of reports of taste and smell disorders.

	Number of Reports	Proportion (%)
Taste or smell disorders	19,342	100
Taste disorders	18,764	97.0
Taste disorders only	17,872	92.4
Smell disorders	1470	7.6
Smell disorders only	578	3.0
Both taste and smell disorders	892	4.6

Taste disorders: Includes all patients with taste disorders regardless of co-existing smell disorders. Taste disorders only: Includes patients with taste disorders without any smell disorders. Smell disorders: Includes all patients with smell disorders regardless of co-existing taste disorders. Smell disorders only: Includes patients with smell disorders without any taste disorders.

**Table 2:** Information on the gender and age of patients using antineoplastic agents.

Gender	Taste and Smell Disorders	Non-Taste and Smell Disorders	*p* (Fisher’s Exact Test)	ROR	95% CI
Male	5391	565,885	0.569	0.990	0.955–1.025
Female	7164	744,229			
**Age**	**Taste and Smell Disorders**	**Non-Taste and Smell Disorders**	***p* (Fisher’s Exact Test)**	**ROR**	**95% CI**
≥70	4561	387,134	<0.0001	1.381	1.332–1.432
<70	8077	946,863			

CI, confidence interval.

**Table 3:** Distribution on volcano plots by drug class.

Medicinal Classification by the ATC Classification	Antineoplastic Agents Likely to Induce Taste and Smell Disorders *#	Number of All Drugs by Drug Class #	Antineoplastic Agents Likely to Induce Taste and Smell Disorders/Number of All Drugs by Drug Class (%)
Alkylating agents (L01A)	1	26	3.85
Antimetabolites (L01B)	1	19	5.26
Plant alkaloids and other natural products (L01C)	1	17	5.88
Cytotoxic antibiotics and related substances (L01D)	2	16	12.5
Protein kinase inhibitors (L01E)	29	73	39.73
Monoclonal antibodies and antibody drug conjugates (L01F)	7	45	15.56
Other antineoplastic agents (L01X)	16	65	24.62

* Antineoplastic agents likely to induce taste and smell disorders as indicated by *p* < 0.05 and ROR > 1 by Fisher’s exact test. # These numbers represent the counts of various antineoplastic agents.

**Table 4:** The *p*-values and RORs by drug category.

Medicinal Classification by ATC Classification	ROR	95% CI
Alkylating agents	0.439	0.410–0.470
Antimetabolites	0.491	0.474–0.509
Plant alkaloids and other natural products	0.710	0.677–0.743
Cytotoxic antibiotics and related substances	0.391	0.356–0.430
Protein kinase inhibitors	2.545	2.498–2.592
Monoclonal antibodies and antibody drug conjugates	0.729	0.703–0.756
Other antineoplastic agents	1.570	1.532–1.609

**Table 5:** Drug clusters.

Cluster 1	Cluster 2	Cluster 3
Avapritinib	Capecitabine	Abemaciclib
Enasidenib	Dasatinib	Afatinib
Enfortumab vedotin	Epirubicin	Alectinib
Entrectinib	Erlotinib	Alpelisib
Erdafitinib	Everolimus	Axitinib
Fedratinib	Mitotane	Bortezomib
Glasdegib	Panitumumab	Bosutinib
Lurbinectedin	Pertuzumab	Cabazitaxel
Pexidartinib	Sorafenib	Cabozantinib
Pralsetinib	Trastuzumab	Crizotinib
Romidepsin	Vorinostat	Daratumumab
Selinexor		Ixazomib
Sonidegib		Lenvatinib
Tazemetostat		Neratinib
Trastuzumab deruxtecan		Nintedanib
Valrubicin		Niraparib
Vismodegib		Olaparib
		Osimertinib
		Palbociclib
		Pazopanib
		Pembrolizumab
		Regorafenib
		Ribociclib
		Rucaparib
		Sunitinib
		Tretinoin
		Tucatinib
		Vemurafenib

**Table 6:** PTs (MedDRA ver. 25.0) and the number of reports for analysis.

PT Name	PT Code	Number of Records
Dysgeusia	10013911	220,220
Ageusia	10001480	70,503
Taste disorder	10082490	34,849
Anosmia	10002653	30,875
Parosmia	10034018	22,325
Hypogeusia	10020989	6926
Hyposmia	10050515	3547
Hallucination, olfactory	10019072	1371
Olfactory nerve disorder	10056388	320
Hypergeusia	10069147	184
Hallucination, gustatory	10019071	157
Gustometry abnormal	10064480	11
Olfactory test abnormal	10062927	9
Olfactory dysfunction	10086567	0

**Table 7:** Cross-tabulation table and formula for ROR.

	Taste and Smell Disorders	Non-Taste and Smell Disorders
Reports with the suspected drug	a	b
All other reports	c	d

ROR, reporting odds ratio [(a/b)/(c/d) = a × d/b × c].

## Data Availability

Data are contained within the article and Supplementary Materials.

## Supplementary Materials

The following supporting information can be downloaded at: https://www.mdpi.com/article/10.3390/ph17091116/s1, Table S1: List of 56 Antineoplastic Agents with Potential Risk of Inducing Taste and Smell Disorders Based on RORs and *p*-values; Table S2: Fisher’s Exact Test Analysis of the Risk of Taste and Smell Disorders Across Different Classes of Antineoplastic Agents; Table S3: lnROR Matrix for Selected Antineoplastic Agents and Taste and Smell Disturbance-Related Adverse Reactions Including Dysgeusia, Ageusia, Taste Disorder, Anosmia, Parosmia, Hypogeusia, and Hyposmia.

## References

[1] Cohen J., Wakefield C.E., Laing D.G. (2016). Smell and taste disorders resulting from cancer and chemotherapy. Curr. Pharm. Des..

[2] Hutton J.L., Baracos V.E., Wismer W.V. (2007). Chemosensory dysfunction is a primary factor in the evolution of declining nutritional status and quality of life in patients with advanced cancer. J. Pain Symptom Manag..

[3] Wickham R.S., Rehwaldt M., Kefer C., Shott S., Abbas K., Glynn-Tucker E., Potter C., Blendowski C. (1999). Taste changes experienced by patients receiving chemotherapy. Oncol. Nurs. Forum.

[4] Comeau T.B., Epstein J.B., Migas C. (2001). Taste and smell dysfunction in patients receiving chemotherapy: A review of current knowledge. Support Care Cancer.

[5] Brisbois T.D., de Kock I.H., Watanabe S.M., Baracos V.E., Wismer W.V. (2011). Characterization of chemosensory alterations in advanced cancer reveals specific chemosensory phenotypes impacting dietary intake and quality of life. J. Pain Symptom Manag..

[6] Tuccori M., Lapi F., Testi A., Ruggiero E., Moretti U., Vannacci A., Bonaiuti R., Antonioli L., Fornai M., Giustarini G. (2011). Drug-induced taste and smell alterations: A case/non-case evaluation of an italian database of spontaneous adverse drug reaction reporting. Drug Saf..

[7] Gamper E.M., Zabernigg A., Wintner L.M., Giesinger J.M., Oberguggenberger A., Kemmler G., Sperner-Unterweger B., Holzner B. (2012). Coming to your senses: Detecting taste and smell alterations in chemotherapy patients. A systematic review. J. Pain Symptom Manag..

[8] Henkin R.I. (1994). Drug-induced taste and smell disorders. Incidence, mechanisms and management related primarily to treatment of sensory receptor dysfunction. Drug Saf..

[9] Schiffman S.S., Zervakis J. (2002). Taste and smell perception in the elderly: Effect of medications and disease. Adv. Food Nutr. Res..

[10] Debbaneh P., McKinnon L., Haidari M., Liang J. (2023). Drug-induced olfactory and gustatory dysfunction: Analysis of FDA adverse events reporting system. Auris Nasus Larynx.

[11] Deems D.A., Doty R.L., Settle R.G., Moore-Gillon V., Shaman P., Mester A.F., Kimmelman C.P., Brightman V.J., Snow J.B. (1991). Smell and taste disorders, a study of 750 patients from the University of Pennsylvania Smell and Taste Center. Arch. Otolaryngol. Head Neck Surg..

[12] Goodspeed R.B., Gent J.F., Catalanotto F.A. (1987). Chemosensory dysfunction. Clinical evaluation results from a taste and smell clinic. Postgrad. Med..

[13] Buttiron Webber T., Briata I.M., DeCensi A., Cevasco I., Paleari L. (2023). Taste and smell disorders in cancer treatment: Results from an integrative rapid systematic review. Int. J. Mol. Sci..

[14] Malta C.E.N., de Lima Martins J.O., Carlos A.C.A.M., Freitas M.O., Magalhães I.A., de Vasconcelos H.C.A., de Lima Silva-Fernandes I.J., de Barros Silva P.G. (2022). Risk factors for dysgeusia during chemotherapy for solid tumors: A retrospective cross-sectional study. Support Care Cancer.

[15] Amézaga J., Alfaro B., Ríos Y., Larraioz A., Ugartemendia G., Urruticoechea A., Tueros I. (2018). Assessing taste and smell alterations in cancer patients undergoing chemotherapy according to treatment. Support Care Cancer.

[16] Zabernigg A., Gamper E.M., Giesinger J.M., Rumpold G., Kemmler G., Gattringer K., Sperner-Unterweger B., Holzner B. (2010). Taste alterations in cancer patients receiving chemotherapy: A neglected side effect?. Oncologist.

[17] Eravcı F.C., Uçar G., Özcan K.M., Çolak M., Ergün Y., Açıkgöz Y., Ikincioğulları A., Uncu D., Dere H.H. (2021). The effect of chemotherapy on olfactory function and mucociliary clearance. Support Care Cancer.

[18] Pugnaloni S., Vignini A., Borroni F., Sabbatinelli J., Alia S., Fabri M., Taus M., Mazzanti L., Berardi R. (2020). Modifications of taste sensitivity in cancer patients: A method for the evaluations of dysgeusia. Support Care Cancer.

[19] Riga M., Chelis L., Papazi T., Danielides V., Katotomichelakis M., Kakolyris S. (2015). Hyposmia: An underestimated and frequent adverse effect of chemotherapy. Support Care Cancer.

[20] Ikeda M., Ikui A., Komiyama A., Kobayashi D., Tanaka M. (2008). Causative factors of taste disorders in the elderly, and therapeutic effects of zinc. J. Laryngol. Otol..

[21] Search for Ethical Drug Information on the Website of the Pharmaceuticals and Medical Devices Agency (PMDA). https://www.pmda.go.jp/PmdaSearch/iyakuSearch/.

[22] Search for drug information on the website of the FDA. https://dailymed.nlm.nih.gov/dailymed/.

[23] van der Werf A., Rovithi M., Langius J.A.E., de van der Schueren M.A.E., Verheul H.M.W. (2017). Insight in taste alterations during treatment with protein kinase inhibitors. Eur. J. Cancer.

[24] Chen L., Xu P., Xiao Q., Chen L., Li S., Jian J.M., Zhong Y.B. (2021). Sunitinib malate inhibits intestinal tumor development in male ApcMin/+ mice by down-regulating inflammation-related factors with suppressing β-cateinin/c-Myc pathway and re-balancing Bcl-6 and Caspase-3. Int. Immunopharmacol..

[25] Lamouille S., Xu J., Derynck R. (2014). Molecular mechanisms of epithelial-mesenchymal transition. Nat. Rev. Mol. Cell Biol..

[26] Zhang W., Zhang H., Wang N., Zhao C., Zhang H., Deng F., Wu N., He Y., Chen X., Zhang J. (2013). Modulation of β-catenin signaling by the inhibitors of MAP kinase, tyrosine kinase, and PI3-kinase pathways. Int. J. Med. Sci..

[27] Barlow L.A. (2015). Progress and renewal in gustation: New insights into taste bud development. Development.

[28] Miura H., Kusakabe Y., Sugiyama C., Kawamatsu M., Ninomiya Y., Motoyama J., Hino A. (2001). Shh and Ptc are associated with taste bud maintenance in the adult mouse. Mech. Dev..

[29] Hummel T., Whitcroft K.L., Rueter G., Haehner A. (2017). Intranasal vitamin A is beneficial in post-infectious olfactory loss. Eur. Arch. Otorhinolaryngol..

[30] Everitt B.S., Landau S., Leese M., Stahl D. (2011). Cluster Analysis.

[31] Jolliffe I.T., Cadima J. (2016). Principal component analysis: A review and recent developments. Philos. Trans. R. Soc. A Math. Phys. Eng. Sci..

[32] Maeda R. (2014). JADER from pharmacovigilance point of view. Jpn. J. Pharmacoepidemiol. Yakuzai Ekigaku.

[33] Noguchi Y., Tachi T., Teramachi H. (2021). Detection algorithms and attentive points of safety signal using spontaneous reporting systems as a clinical data source. Brief. Bioinform..

[34] Pariente A., Gregoire F., Fourrier-Reglat A., Haramburu F., Moore N. (2007). Impact of safety alerts on measures of disproportionality in spontaneous reporting databases: The notoriety bias. Drug Saf..

[35] Hartnell N.R., Wilson J.P. (2004). Replication of the Weber effect using postmarketing adverse event reports voluntarily submitted to the United States Food and Drug Administration. Pharmacotherapy.

[36] Wang H.W., Hochberg A.M., Pearson R.K., Hauben M. (2010). An experimental investigation of masking in the US FDA adverse event reporting system database. Drug Saf..

[37] Pariente A., Avillach P., Salvo F., Thiessard F., Miremont-Salamé G., Fourrier-Reglat A., Haramburu F., Bégaud B., Moore N., Association Française des Centres Régionaux de Pharmacovigilance (CRPV) (2012). Effect of competition bias in safety signal generation: Analysis of a research database of spontaneous reports in France. Drug Saf..

[38] Poleksic A., Xie L. (2019). Database of adverse events associated with drugs and drug combinations. Sci. Rep..

[39] FDA Adverse Event Reporting System (FAERS). https://www.fda.gov/drugs/drug-approvals-and-databases/fda-adverse-event-reporting-system-faers.

[40] Lumini A., Nanni L. (2018). Convolutional neural networks for ATC classification. Curr. Pharm. Des..

[41] MedDRA Japanese Maintenance Organization. https://www.meddra.org/.

[42] Watanabe H., Matsushita Y., Watanabe A., Maeda T., Nukui K., Ogawa Y., Sawa J., Maeda H. (2004). Early detection of important safety information. Recent methods for signal detection. Jpn. J. Biomet..

[43] Ohyama K., Sugiura M. (2018). Evaluation of the association between topical prostaglandin F2α analogs and asthma using the JADER database: Comparison with β-blockers. Yakugaku Zasshi.

[44] Greenland S., Schwartzbaum J.A., Finkle W.D. (2000). Problems due to small samples and sparse data in conditional logistic regression analysis. Am. J. Epidemiol..

[45] Gravel C.A., Douros A. (2023). Considerations on the use of different comparators in pharmacovigilance: A methodological review. Br. J. Clin. Pharmacol..

[46] Hosoya R., Uesawa Y., Ishii-Nozawa R., Kagaya H. (2017). Analysis of factors associated with hiccups based on the Japanese Adverse Drug Event Report database. PLoS ONE.

[47] Kawabe A., Uesawa Y. (2023). Analysis of corticosteroid-induced glaucoma using the Japanese adverse drug event reporting database. Pharmaceuticals.

[48] Okunaka M., Kano D., Matsui R., Kawasaki T., Uesawa Y. (2021). Comprehensive analysis of chemotherapeutic agents that induce infectious neutropenia. Pharmaceuticals.

[49] Kan Y., Nagai J., Uesawa Y. (2021). Evaluation of antibiotic-induced taste and smell disorders using the FDA adverse event reporting system database. Sci. Rep..

[50] Nakao Y., Asada M., Uesawa Y. (2023). Comprehensive study of drug-induced pruritus based on adverse drug reaction report database. Pharmaceuticals.

[51] Chen J.J., Wang S.J., Tsai C.A., Lin C.J. (2007). Selection of differentially expressed genes in microarray data analysis. Pharmacogenom. J..

[52] Van Puijenbroek E.P., Bate A., Leufkens H.G.M., Lindquist M., Orre R., Egberts A.C. (2002). A comparison of measures of disproportionality for signal detection in spontaneous reporting systems for adverse drug reactions. Pharmacoepidemiol. Drug. Saf..

